# Consolidation Modeling during Thermoforming of Thermoplastic Composite Prepregs

**DOI:** 10.3390/ma12182853

**Published:** 2019-09-04

**Authors:** Hu Xiong, Nahiène Hamila, Philippe Boisse

**Affiliations:** LaMCoS CNRS, INSA-Lyon, Université de Lyon, F-69621 Lyon, France

**Keywords:** forming, thermoplastic, prepreg, consolidation, process simulation, finite element method

## Abstract

This article describes the modeling of the compaction/consolidation behavior of thermoplastic composite prepregs during the thermoforming process. The proposed model is principally based on a generalized Maxwell approach. Within a hyperelastic framework, viscoelasticity is introduced for the compaction mode in addition to the in-plane shearing mode by taking into account the influence of the resin and its flow during consolidation. To reveal the evolution of the consolidation level, which reflects the number of voids in the composite, an intimate contact model was used during the process. The model was characterized by a compaction test at a high temperature. It was implemented into a recently developed prismatic solid-shell finite element. The analysis of the thermoforming of a double dome demonstrated the relevance of the consolidation computation in determining the process parameters leading to a composite part free of voids.

## 1. Introduction

Continuous fiber-reinforced prepreg thermoplastic composites are of interest to the automotive industry [1,2,3] and by using thermoforming, short cycle times are made possible. This manufacturing technique can be easily automated and can be achieved thanks to technologies that are close to those of metal sheet forming. Furthermore, the higher impact resistance and ductility of thermoplastic prepregs, as well as their reparability and recyclability, offer great competitiveness over composites with thermosetting matrices.

**Figure 1:**
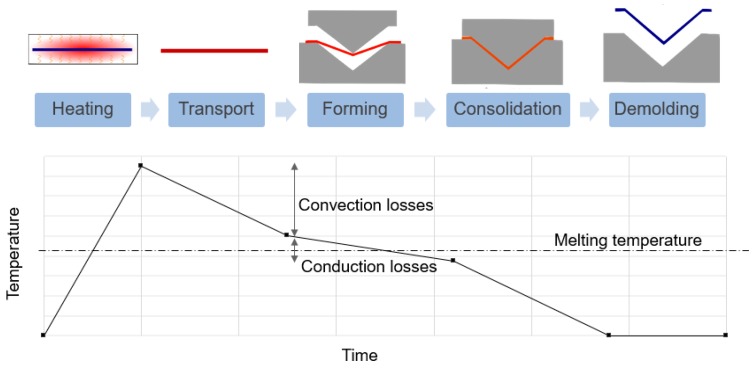
Principal steps of the thermoplastic thermoforming process.

As illustrated in Figure 1, the complete thermoforming process consists of five stages. The thermoplastic prepreg blank is preliminarily heated slightly over the fusion temperature of the matrix, then quickly transported to a mold. After shaping of the part, a pressure is applied and maintained to consolidate the composite, and the temperature is lowered for crystallization. Subsequently, the final part is obtained by demolding. The thermoforming process is complex considering the multidisciplinary problems, such as the thermal, mechanical and physical aspects [4,5,6,7]. For the purpose of producing a finished product with a determined cost and quality, the manufacturing process must be carefully optimized with appropriate processing conditions. Virtual manufacturing by numerical simulations has become an essential approach to replace the traditional (and costly) trial and error processes [8,9,10]. In view of this, some investigators have created their proper models by considering the processing parameters (temperature, pressure), the architecture of the composite structure (stacking, multiple plies, etc.), and the defects (wrinkles, porosity, distortion, micro fractures, etc.) during the thermoforming process. Simulation methods have thus been developed for the forming stage (draping) [5,8,10,11,12,13,14,15,16,17,18].

The consolidation phase is very important for the quality of the composite part. During this step, pressure is applied to the formed part that leads to compaction to ensure a good cohesion of the matrix by removing the inter- and intra-ply porosities. Modeling of the consolidation step thus helps to predict the time consumption and defect content under the given process parameters, i.e., temperature, pressure, etc. The objective of this article is to propose a simulation of the prepreg thermoforming process including the consolidation phase in order to determine the time and pressure required for consolidation of a composite part of any geometry.

In the recent past, different aspects of consolidation for thermoplastic and thermosetting composites during manufacturing have been addressed. These include automated lay-up [19,20,21], VARTM process (Vacuum Assisted Resin Transfer Molding) [22], autoclave [23], and compression flow molding [24,25], all of which focused on experimental investigations. Furthermore, Helmus et al. proposed both a stochastic approach [26] and an air evacuation-coupled impregnation model [27] for out-of-autoclave prepreg consolidation. In the framework of the finite element method, a two-dimensional thermal model [28] and a constitutive model [29] have been developed for composite consolidation during a filament winding process. Wrinkling is one of the major failure mechanisms [30] and can be induced by consolidation. Some authors have analyzed the generation of wrinkling during consolidation both in experimental studies and finite element simulations [31,32].

This resin flow is governed by Darcy’s law [33,34], coupled with fiber bed compaction behavior, which is assumed to be an elastic porous medium with incompressible and inextensible fibers. The compaction behavior is described by the fiber volume fracture as a function of the pressure [35,36,37,38,39,40]. Another typical resin flow is transverse squeeze flow to describe the viscous incompressible thermoplastic fluid moving transversely together with the fibers. The fluid is modeled according to either a Newtonian [41,42,43] or non-Newtonian [44] behavior or both [45]. Taking into account the two flow types, Belnoue et al. [46] recently proposed a viscoelastic model in terms of strain- and strain rate-dependent behaviors for uncured toughened prepreg systems. Besides the resin flow model, many authors have combined other models to reveal the consolidation or compaction behavior. These include a fiber bed elastic model [35,36,38,39,47,48,49], an impregnation model [49,50,51,52], and an interply cohesion model [51,52,53].

Based on the aforementioned issues, the present work concerns both the matrix’s viscosity, the elasticity of the fiber bed and the interply cohesion. Based on the experimental and analytical studies of [37,46,54,55], Section 2 proposes a viscoelastic model combining the elastic behavior of dry fiber reinforcements and the viscous behavior of the resin. For the purpose of evaluating the consolidation level, an intimate contact model is introduced in Section 3 by considering the uneven ply surface of thermoplastic prepregs. This model makes it possible to compute the evolution of the consolidation (porosity closure) level and the thickness as a function of the pressure and temperature during manufacturing. Section 4 presents a simple experimental compaction test at a high temperature to identify the parameters of the proposed models. Finally, the compaction test and a double dome thermoforming process were simulated by the models. A list of symbols is given in Appendix B.

## 2. Viscoelastic Characterization for Compaction Behavior

Accurate prediction models for unique and complex material behaviors remain a key challenge of process simulation. In many cases, both elastic and viscous responses are outlined to describe fiber-reinforced epoxy composites, especially for intra-ply mechanisms [11,17,18,56,57,58]. When 3D states of stress are applied during processing, such as the consolidation investigated in the current work, a through-the-thickness mechanical behavior needs to be considered. Based on a previous visco-hyperelastic model for shells [11], the transverse compaction behavior within the viscoelastic response is addressed by taking into account the rheology of the composites, including the fiber bed and resin flow.

The definition of a hyperelastic constitutive model is derived from the Clausius–Duhem inequality equation with an assumption of no dissipated energy. The constitutive equation of the model gives the 2nd Piola–Kirchoff stress tensor $\underset{=}{S}$ derived from an internal potential energy w (per undeformed unit volume) that depends on the right Cauchy–Green strain tensor $\underset{=}{C}$ [59]:

(1) $$\underline{\underline{S}}=2\frac{\partial w(\underline{\underline{C}})}{\partial \underline{\underline{C}}}$$

For hyperelastic materials with an anisotropic response, the anisotropy is simply embodied in the potential w, providing a natural framework for a frame-invariant formulation [60].

(2) $$w(\underline{\underline{C}})={w(I}_{1},I_{2},I_{3}\ldots ,I_{n})$$

The hyperelastic laws for a 2D fabric [56] and 3D interlock [61,62,63] were recently developed. This approach, inspired by the work of Criscione [64], involves a multiplicative decomposition of the deformation gradient $\underset{=}{F}$. Each term of this decomposition linking to the classical invariants are characterized by a “physical” invariant $I_{n}$. The advantage consists in decoupling the deformation modes, after which they are investigated separately.

**Figure 2:**
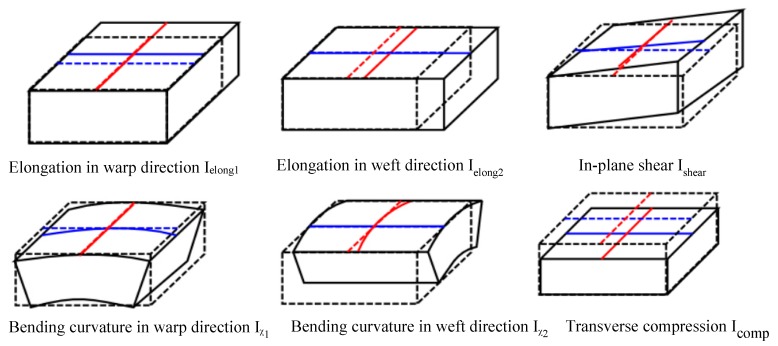
Deformation modes for woven reinforcement within a hyperelastic framework.

A thin shell structure is usually assumed for the thermoplastic prepreg composites. The considered deformation modes are membrane (including elongation and in-plane shear) and bending. When the through-the-thickness behavior becomes predominant during forming, the transverse compression deformation mode is considered. Figure 2 presents all of the considered deformation modes with their respective physical invariants.

The volume strain energy is the sum of the different strain energies that are assumed to depend only on the corresponding physical invariants [11,62].

(3) $$w=w_{\mathrm{elong}1}\left(I_{\mathrm{elong}1}\right)+w_{\mathrm{elong}2}\left(I_{\mathrm{elong}2}\right)+w_{\mathrm{shear}}\left(I_{\mathrm{shear}}\right)+w_{\mathrm{bend}1}\left(I_{\chi 1}\right)+w_{\mathrm{bend}2}\left(I_{\chi 2}\right)+w_{\mathrm{comp}}\left(I_{\mathrm{comp}}\right)$$

In the present study on thermoplastic prepregs, only the in-plane shear and compaction viscosities were taken into account. The tension and bending behavior were assumed to be elastic [65]. The viscoelastic model for compaction is detailed in Section 2.1. All the other strain potential energies, taking the thermal and viscous behaviors into account or not, are provided in Appendix A.

### 2.1. Viscoelastic Model for Compaction Behavior

**Figure 3:**
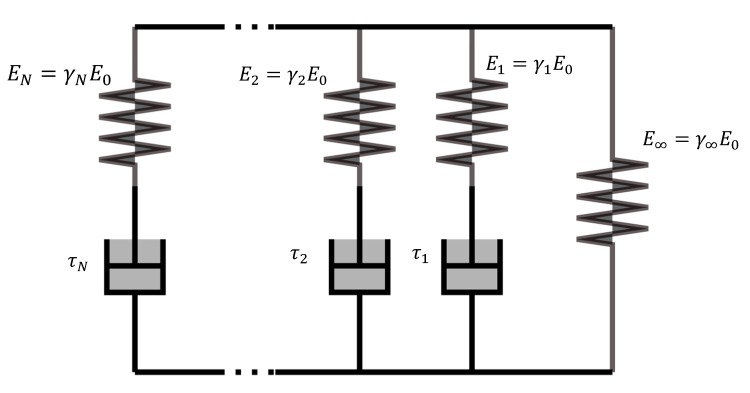
Viscoelastic model with springs and dashpots.

As discussed previously, some authors have described a viscoelastic response for the compaction behavior for both dry and wet fibrous materials [36,54,55]. During the forming process, the presence of the molten matrix and its flow phenomena, especially the resin percolation and the transverse squeezing flow, highlight the viscous proportion for the mechanical consolidation behavior. In view of the viscous description for the matrix flow and the fiber bed’s elastic behavior, a viscoelastic model for the consolidation behavior was proposed in the framework of Guzman’s thermo-hyper-viscoelastic constitutive law [11,12] for thin thermoplastic prepreg composites. In this model (shown in Figure 3), a single spring element is placed in parallel with N Maxwell elements, and the elastic stiffness for all the springs is represented by the Young modulus $E_{\infty }$ and $E_{i}$. These viscous effects are modeled by N dashpots with a constant viscosity $\eta_{i}$, a set of stress-like internal variables ${\underline{\underline{Q_{i}}}}^{comp}$ are completed for the free energy function of the compaction deformation mode (the *comp* index is relative to compaction) [11,66]:

(4) $$\psi^{comp}(\underline{\underline{C}},{\underline{\underline{Q_{i}}}}^{comp})=w_{comp}(I_{comp})-\frac{1}{2}\sum_{i=1}^{N}(\underline{\underline{C}}:{\underline{\underline{Q_{i}}}}^{comp})$$

The first elastic part indicates the compaction behavior of dry reinforcement, whose potential energy is formulated with a fifth-order polynomial function:

(5) $$w_{comp}=\sum_{i=1}^{5}D_{k}{\left(I_{comp}\right)}^{k}$$

Clausius Duhem’s inequality introduced into Equation (4) derives the compression deformation-related stress tensor by the following expression [11,66]:

(6) $${\underline{\underline{S}}}_{comp}=2\frac{\partial w_{comp}(I_{comp})}{\partial \underline{\underline{C}}}-\sum_{i=1}^{N}{\underline{\underline{Q_{i}}}}^{comp}$$

For a generalized-Maxwell model, Simo and Hughes [66] provided detailed evolution equations for the internal variables ${\underline{\underline{Q_{i}}}}^{comp}$ in the present work) and their algorithmic solutions ${\underline{\underline{H}}}_{i}$ at current time *t*_*n*+1_:

(7) $${\underline{\underline{H}}}_{i}(t_{n+1})=\exp(-\frac{\Delta t_{n}}{\tau_{i}}){\underline{\underline{H}}}_{i}(t_{n})+\exp(-\frac{\Delta t_{n}}{2\tau_{i}})({\underline{\underline{S}}}_{n+1}^{0}-{\underline{\underline{S}}}_{n}^{0})$$

Here, ${\underline{\underline{S}}}_{n+1}^{0}$ is the stress tensor attributed by the pure elastic part (superscript 0) at the time *t*_*n*+1_ (subscript *n*+1). *τ*_i_ denotes the relaxation time.

Hence, the stress tensor from Equation (6) is consequently computed as a function of the internal variables:

(8) $${\underline{\underline{S}}}_{comp}(t_{n+1})=\gamma_{\infty }{\underline{\underline{S}}}_{n+1}^{0}+\sum_{i=1}^{N}\gamma_{i}{\underline{\underline{H}}}_{i}(t_{n+1})$$

Within the relative weights $\gamma_{i}=E_{i}/E_{0}\in [0,1],\;\gamma_{\infty }=1-\sum_{i=1}^{N}\gamma_{i}$.The viscoelastic model to describe transverse compression behavior is eventually established. The related model parameters such as D_k_, τ_i_, and γ_i_, could be calibrated by compaction tests at a high temperature. This is presented in Section 4.

## 3. Prediction of the Consolidation Level

### 3.1. Intimate Contact Model

Consolidation induces complex phenomena during thermoplastic composite thermoforming. These phenomena have been investigated at three levels. Most of the studies were focused on intimate contact between layers, interlaminar bonding (autohesion), impregnation of the fibers, as well as molecular diffusion at microscopic and molecular levels [52,53,67,68]. Moreover, macroscopic analysis involves the laminate properties, such as fiber volume fraction, mechanical properties and void content, related to the processing parameters. In this work, the macro level was considered for the purpose of predicting the consolidation level through the global composite part during thermoforming.

**Figure 4:**
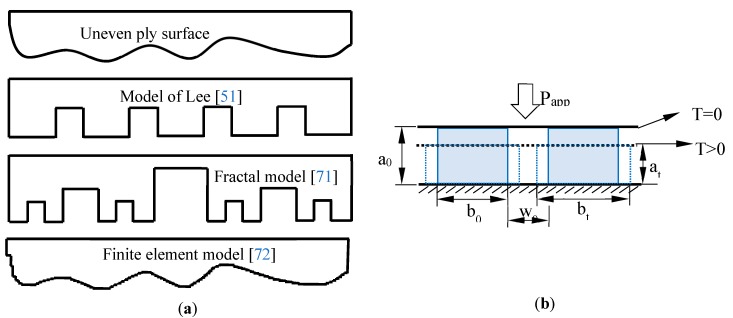
Intimate contact model: (**a**) Different approaches; (**b**) Discretization by regular rectangular elements.

The intimate contact model has been widely described in the literature due to its simplicity when it comes to representing the coalescence between two adjacent ply surfaces during the manufacturing of thermoplastic composites. In this model, a degree of intimate contact, corresponding to the coalescence level, is defined geometrically from the initial roughness of the ply surface. In view of the description of the surface, several related models have been developed. The most popular model is the approach proposed by Lee et al. [51]. In their work, the roughness of the ply surface was modeled by a series of periodic rectangular asperities (Figure 4a). Several authors have used this model for their investigations [52,53,68,69,70] and assumed constant pressure and temperature during the intimate contact process. With the aim of getting closer to reality, other options like the fractal model [71] and the finite element model [72] show good correlation with the original model and experimental results.

### 3.2. A Time Discrete Approach

Following the investigation of Lee and Springer [51], a series of identical rectangles were employed to signify the initial ply surfaces with irregularities (Figure 4b). Here, the matrix was represented by blue solid rectangles of dimension $a_{0}b_{0}$ (0 refers to the initial state), and the remaining elements $w_{0}b_{0}$ denoted the void defects in the composite part. As the processing force or pressure was applied, the resin rectangles spread over the interface with b0 extending to b (at an actual time state t). Simultaneously, the void width dwindled to w. During the rheology of this mechanism, the degree of intimate contact D was defined as [51]:

(9) $$D=\frac{b}{w_{0}+b_{0}}$$

During processing, hypotheses were made. The width of the smallest repeated volume unit (one matrix element + one void element) b+w was assumed to be constant, and the matrix was considered as an incompressible material. Hence:

(10) $$w_{0}+b_{0}=w+b,\;a_{0}b_{0}=ab$$

Next, the law of conservation of mass was taken into account. A laminar Newtonian flow was assumed for the resin elements, and the velocity field could be described by Poiseuille’s law [73]. Based on these assumptions, Lee and Springer [51] finally obtained an ordinary differential equation governing the ply height a with the process parameters:

(11) $$\frac{1}{5a^{5}}=\frac{p_{app}\left(b_{0}+w_{0}\right)}{a_{0}^{3}b_{0}^{3}\eta }t+C_{3}]$$

Here, the viscosity *η* was assumed constant in the isothermal case. *C*_3_ was a constant related to the initial condition.

As the pressure *P* is a function of the time *t*, the evolution of the pressure is discretized with n increments of time Δ*t* (small enough). The time returns to 0 at the beginning of each time increment. Meanwhile, the applied pressure *p*_app n_ is presumed constant, and the following relations are assumed:

*t* = 0, *a* = *a_n_*,  *t* = Δ*t*, *a* = *a*_n+1_ (12)

At time *t* = 0, $C_{3}=\frac{1}{5a_{n}^{5}}$. The width after the n^th^ time increment is

(13) $$a_{n+1}={\left[\frac{5p_{app\;n}\left(b+w_{0}\right)\Delta t}{a_{0}^{3}b_{0}^{3}\eta }+\frac{1}{a_{n}^{5}}\right]}^{-1/5}$$

Substituting Equation (10) into Equation (9), the actual degree of intimate contact is rewritten as:

(14) $$D_{n+1}=\frac{a_{0}/a_{n+1}}{1+w_{0}/b_{0}}$$

For simplification, *k*_1_ and *k*_2_ are introduced with $k_{1}=\frac{a_{0}}{b_{0}},k_{2}=\frac{w_{0}}{b_{0}}.$

Combining the previous equations yields:

(15) $$a_{n+1}={\left[\frac{5p_{app\;n}\left(1+k\right)k_{1}^{2}\Delta t}{a_{0}^{5}\eta }+\frac{1}{a_{n}^{5}}\right]}^{-1/5}\;\;D_{n+1}=\frac{a_{n}/a_{n+1}}{1+k_{2}}$$

Hence, with the time discrete approach, the intimate contact model is expressed under non-constant pressure. The degree of intimate contact evolves instantaneously as a function of pressure *P_app_*, temperature *T*, consolidation time *t*, and material parameters, such as fiber-matrix mixture viscosity *η*, and the geometric ratios *k*_1_ and *k*_2_, which can be also identified from a compaction test. It should be noticed that the developed approach is equal to the Lee-Springer model when the pressure is assumed constant during processing.

### 3.3. Remark on Consolidation and Crystallisation

After the forming process at a high temperature, the composite is cooled and undergoes crystallisation of the resin to obtain the hardened composite. Consolidation and crystallization are two important aspects that are related but nevertheless different. Consolidation is achieved if the composite has no voids. This is the purpose of this study. At the end of a thermoforming process, this absence of voids (or a sufficiently low void fraction) is necessary to obtain good mechanical properties of the composite part. This consolidation is analyzed here using Lee and Springer’s [51] model, which determines the degree of intimate contact.

The crystallization of the semi-crystalline thermoplastic matrix is essential for the properties of the final composite [74,75]. Cooling conditions (cooling rate and pressure) affect the crystallization and quality of the composite [76,77,78,79,80]. This cooling phase is of great importance, but is not directly addressed in this work. This one proposes a method of simulation of the thermoforming process and the calculation of a consolidation indicator (degree of intimate contact).

## 4. Compaction Tests at a High Temperature

Compaction can be used to identify these models [37,46,47,81]. Based on the method presented in [47], the compaction test at a high temperature is carried out to identify the parameters both for the proposed viscoelastic compaction model and the time-discretized intimate contact model. Furthermore, the obtained experimental results are compared to those of the simulations during the final validation.

### 4.1. Material

The material analyzed in this paper was composed of a 2 × 2 woven twill reinforcement and a thermoplastic resin (polyamide 6-6, PA 6-6). The material plates containing four layers were pre-impregnated and produced by Solvay. The melting temperature was set at 262 °C. The principal material properties are detailed in Table 1.

### 4.2. Experimental Set-up

**Figure 5:**
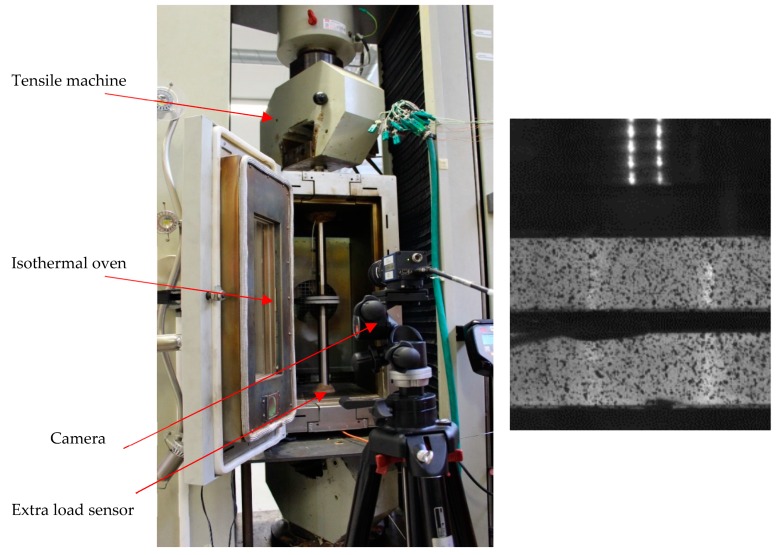
High-temperature compaction test device.

Figure 5 shows the schematic of the compaction test device (Tensile machine RSA 250, Schenck, Darmstadt, Germany) at a high temperature for thermoplastic prepregs. Two metallic plates linked to a tensile test machine were used to compress the specimen with dimensions of 60 mm × 60 mm. The device was caged in an isothermal oven. Besides the internal thermocouple in the oven, three additional thermocouples were employed in order to precisely follow the evolution of the temperature over time: one was inserted in the cross-section of the material (through a small hole with a 0.5-mm diameter and 2-mm depth), and the other two were fixed in the two metallic plates. Prior to the compaction step, the system was heated to the desired temperature. The thermal gradient was assumed to be negligible inside the specimen during the tests. As the mechanical system of the tensile machine was equipped with damping, its own transverse displacement sensor seemed insufficiently precise. Instead, a digital image correlation (DIC) system was used to correctly determine the compression displacement through the images taken by a CCD camera (Camera Mega+, Kodak, Rochester, NY, USA).Furthermore, compression measurements were provided by an extra load sensor placed at the bottom of the support.

### 4.3. Experimental Conditions

It should be noted that the exposition time of the material must be controlled and limited because of the risk of matrix oxidation at a high temperature. Before introducing the specimen, the system was rapidly heated to about 300 °C. Subsequently, the sample was placed in the machine, causing the temperature to decrease below 300 °C. For this reason, another 5 min of heating was needed to obtain a more homogenous temperature field (about 300 °C in accordance with the thermoforming process) for the whole equipment, especially for the sample. Then, cooling was performed gradually to the desired test temperature, and followed by the start-up system to activate the compression and camera recording. In order to model the compression behavior during the process at different temperature levels, a series of tests were performed over the range of 260–300 °C, while the compaction speed was fixed at 0.2 mm/min. A maximum load value was set to 8000 N as the test limit. Once this value was achieved, the loading and image recording system became deactivated. Finally, the system was cooled by opening the mesh and resetting the top plate to its initial position.

### 4.4. Experimental Results

**Figure 6:**
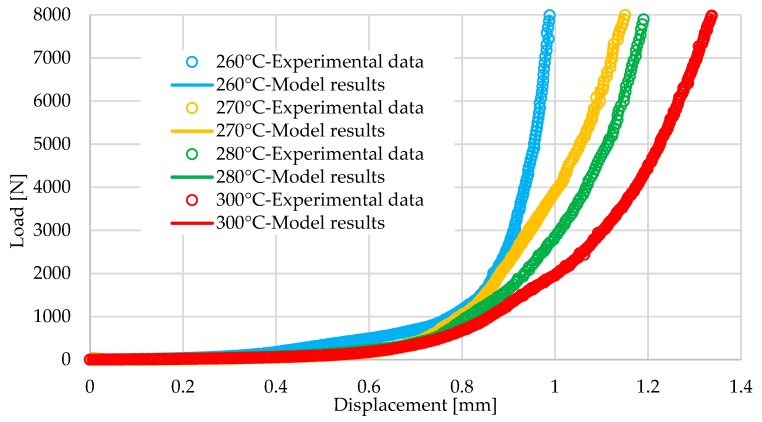
Comparison between experimental and optimal results for a 2 × 2 twill in carbon/PA 6-6 prepregs.

Figure 6 shows the load versus displacement curves at different temperatures. One can notice that the compaction stiffness decreased when the temperature increased. Based on the experimental results, an inverse method (the Levenberg-Marquardt method) [82] was employed to optimize the parameters of the proposed model over the temperature range 260–300 °C. The calibrated parameters are given in Table 2 and Table 3. Here, their optimal loads at different temperatures are compared with the experimental loads demonstrated in Figure 6.

## 5. F.E. Simulations of Thermoforming

### 5.1. Prismatic Solid Shell Finite Element Model

**Figure 7:**
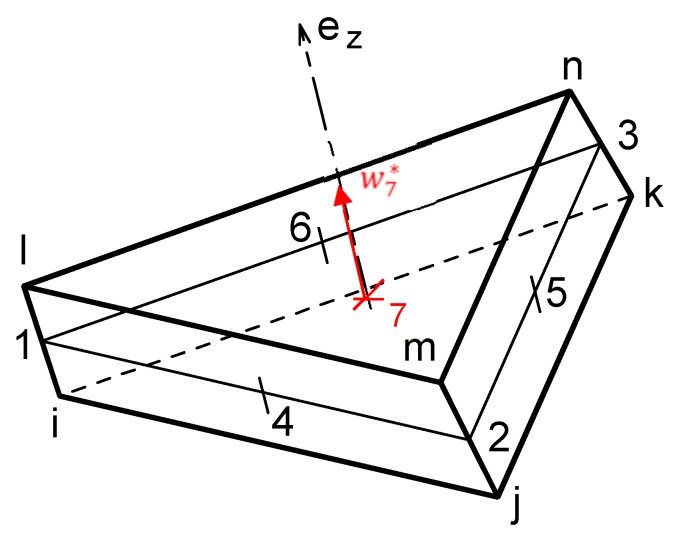
Seven-node prismatic solid-shell element for the consolidation simulation.

Many mechanical approaches exist for modeling of textile composite forming at the macroscopic scale [83,84], including discrete approaches [85,86], continuous methods [5,8,11,12,18,46], and semi-discrete techniques [87,88,89]. All of them focus on the in-plane and bending behaviors during the forming step, whereas the through-the-thickness behavior is ignored with the assumption of a thin plate theory. For the purpose of thin/thick shell analysis by considering the evolution of the consolidation effect during the thermoforming process of the prepreg composites, a new seven-node prismatic solid-shell finite element approach was recently developed [90]. As shown in Figure 7, the new prismatic finite element possesses seven nodes: six (i,j,k,l,m,n) apical nodes and an extra one at the center, and 19 degrees of freedom (DOF) of translation: three for each apex, one along the thickness at the seventh center node.

Equation (16) shows the virtual work theorem for any virtual displacement field δu, in which each virtual work (external, internal and inertial) is denoted by $W_{\mathrm{ext}},{\;W}_{\mathrm{int}},{\;\mathrm{and}\;W}_{\mathrm{acc}}$, respectively. Three deformation components, i.e., bending, membrane, and pinching, are described separately for the internal virtual work.

(16) $$W_{\mathrm{ext}}\left(u,\delta u\right)-W_{\mathrm{int}}\left(u,\delta u\right)=W_{\mathrm{acc}}\left(u,\delta u\right)$$

with

(17) $$W_{\mathrm{int}}\left(u,\delta u\right)=W_{\mathrm{int}}^{\mathrm{bending}}\left(u,\delta u\right)+W_{\mathrm{int}}^{\mathrm{membrane}}\left(u,\delta u\right)+W_{\mathrm{int}}^{\mathrm{pinching}}\left(u,\delta u\right)$$

The virtual work theorem equation can be expressed by finite element interpolation, which leads to [91]:

(18) $$M\ddot{u}+F_{\mathrm{int}}^{\mathrm{bending}}+F_{\mathrm{int}}^{\mathrm{membrane}}+F_{\mathrm{int}}^{\mathrm{pinching}}-F_{\mathrm{ext}}=0$$

where the mass matrix is represented by M, $\ddot{u}$ is the nodal acceleration vector, and $F_{\mathrm{ext}}$ is the single column matrix of the exterior load. The single column matrix for each internal load, $F_{\mathrm{int}}^{\mathrm{bending}}$, $F_{\mathrm{int}}^{\mathrm{membrane}}$, and $F_{\mathrm{int}}^{\mathrm{pinching}}$, can be simply written by $F_{e,\mathrm{int}}^{\alpha }$ [91]:

(19) $$F_{e,\mathrm{int}}^{\alpha }=\int_{\Omega_{e}}^{}B^{\alpha }{}^{T}\sigma^{\alpha }d\Omega$$

Here, α refers to three considered deformation modes (bending, membrane, and pinching), and $\sigma^{\alpha }$ is the related Cauchy stress. For each attributed mode in the introduced prismatic solid shell element, the conventional DKT6 shell element offers the bending part, while the formulation of the CST shell element provides membrane formulations. In the aspect of pinching, the enhanced assumed strain (EAS) method is carried out within the extra displacement DOF imported by the seventh node. As a result, a linear variation of stresses in thickness is obtained and the thickness locking is avoided. Because a reduced integration (RI) scheme is used, an hourglass stabilization (HS) procedure is performed to correct the element’s rank deficiency for pinching, while transverse shear deformations are calculated by the assumed strain (AS) method to prevent specific zero energy modes. Based on the above ideas, the strain interpolation matrix $B^{\alpha }$ related to mode α was formulated in the work of Xiong et al. [90]. This solid shell element has the same efficiency as the shell elements for thin parts, but also allows the use of a complete 3D constitutive law (Section 2) within the through-the-thickness behavior.

**Figure 8:**
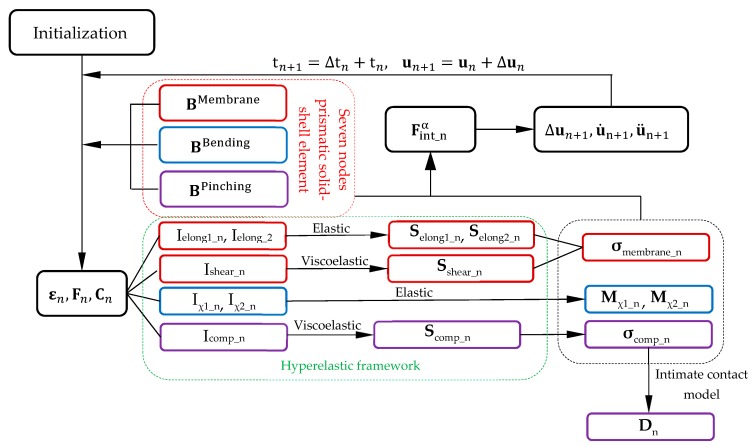
Flowchart of the finite element analysis of the degree of consolidation D_n_.

The presented 3D visco-hyperelastic constitutive model for thermoplastic prepreg composites and the time discrete intimate contact model described above was implanted in the finite element code Plasfib [92] based on an explicit dynamic approach. The prismatic solid shell element presented in Section 5.1 is used in this simulation. The flowchart of the finite element analysis of the degree of consolidation is carried out according to Figure 8.

### 5.2. Simulation of Compaction Tests

**Figure 9:**
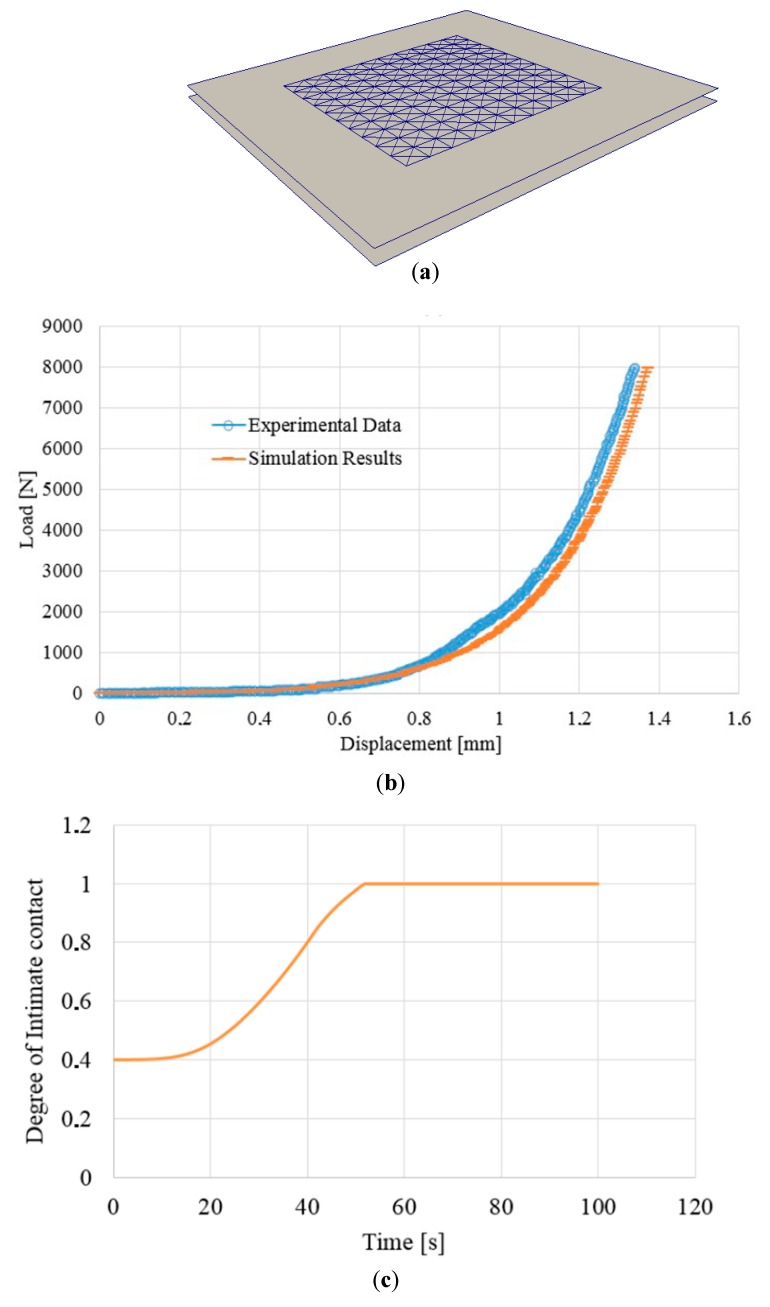
Simulation of a compaction test at 300 °C. (**a**) Meshed structure (composite blank in the middle); (**b**) Simulated and experimental load versus displacement curves; (**c**) Evolution of the degree of intimate contact during the simulation.

Figure 9a demonstrates the meshed geometry of the compaction test, conforming to the experimental set-up. The metallic compression plates were modeled by two rigid plates, within the squared plate (60 mm × 60 mm × 2.5 mm) placed inside. The mechanical properties for the studied thermoplastic composites calibrated at 300 °C were used during this numerical compaction test. The bottom plate was fixed, while the top one was subject to a displacement of 1.4 mm in the downward vertical direction. The speed of the displacement loading was 2 mm/min, which corresponds to the experimental loading speed.

Figure 9b depicts the comparison between the simulated load and the experimental load as a function of displacement. It was shown that the simulation results were in good agreement with the experiment. In addition, the evolution of the degree of intimate contact D during the compaction test is represented in Figure 9c, where the degree of intimate contact equated 0.8 at about 40 s as the maximum load was achieved, and by keeping this maximum value within approx. 10 s, the complete consolidation (D = 1) could take place.

### 5.3. Thermoforming Simulation of a Double Dome

As a forming benchmark example owing to its double-curved structure, the double dome test enables comparative investigations of the existing models both for continuous dry and pre-impregnated reinforcements at room temperature or high temperatures [14,93,94,95,96,97,98]. It is also conducted to investigate various relevant aspects such as the effect of fiber orientations due to the process on the in-service behavior properties of the composite [96].

**Figure 10:**
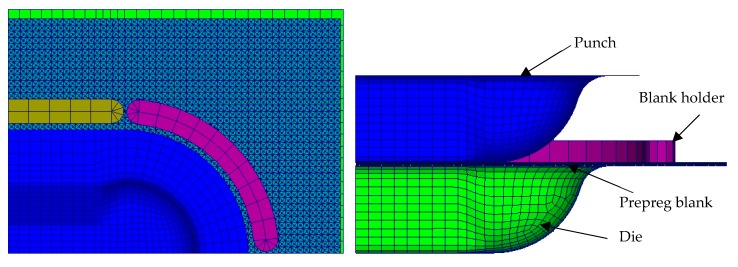
Double dome structure (geometry and mesh).

Figure 10 only shows one quarter of the process structure for symmetric reasons. The rectangular pre-impregnated blank, with dimensions of 270 mm × 190 mm × 2 mm, was placed between the punch and the die. Moreover, six blank holders were added to maintain the blank in contact. Within the related properties identified at different temperatures, the material of the studied carbon-twill/PA 6-6 thermoplastic prepregs was applied and orientated in 0/90° directions. Isothermal and homogeneous distribution assumptions were made during the simulations, and the temperature was fixed at 300 °C, which corresponds to the manufacturing temperature. The objective of this simulation was to investigate the evolution of consolidation during the manufacturing process including the thermoforming and consolidation steps.

**Figure 11:**
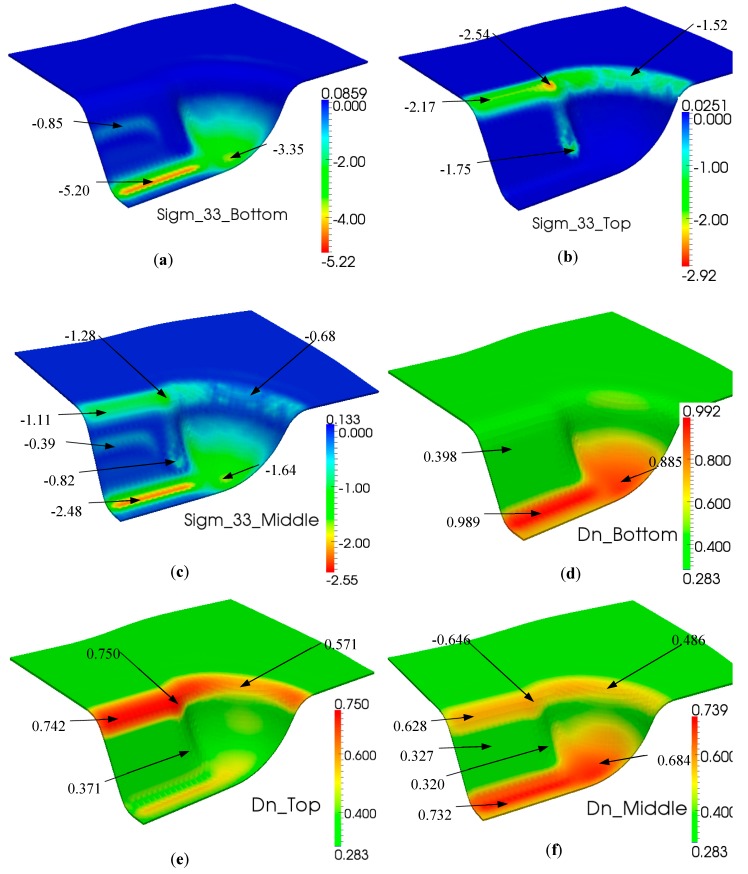
Simulation results during the forming step of a double dome test. Normal stress σ_33_ in (**a**–**c**) and intimate contact Dn in (**d**–**f**).

Firstly, simulation of the forming stage was launched subject to a vertical displacement of the punch. The total displacement was 60 mm and its rate was set to 240 mm/min. The through-the-thickness behavior could be simultaneously analyzed all along the simulation, and Figure 11a–c give the final distributions of the transverse stress σ_33_ in the bottom, top and middle faces at the end of this step. Moreover, the degree of intimate contact Dn, which represents the consolidation level, is depicted in Figure 11d–f for the bottom, top and middle faces, respectively.

**Figure 12:**
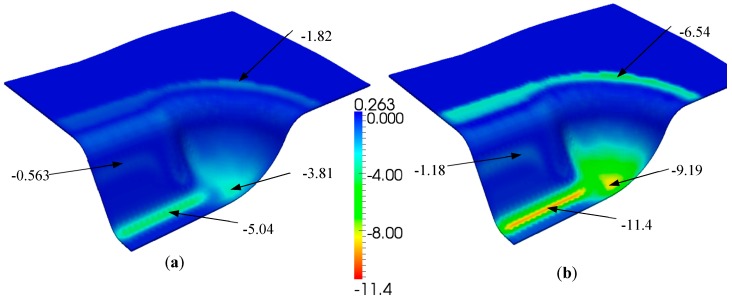
Distribution of transverse stress σ_33_ (middle face) during the consolidation process: (**a**) σ_33_ (MPa) for Δu = 0.2 mm; (**b**) σ_33_ (MPa) for Δu = 0.5 mm.

As most of the deformed piece did not achieve the completed consolidation level (Dn = 1) after the forming step, additional time consumption or extra pressure was required. Due to the cost of time, a reasonable pressure was first considered. In the finite element simulation case, extra compression displacement on the punch was applied, while the equivalent load integrated through the internal forces of each element was obtained. In order to investigate the influence of compaction, a set of simulations was performed within two compressed displacements: Δu = 0.2 mm and 0.5 mm. Consequently, the equivalent loads Fn were increased from 2.85 kN (at the end of the forming step) to Fn = 4.95 kN and 10.44 kN, respectively. Their distributions of transverse stress σ_33_ in the middle face are shown in Figure 12.

**Figure 13:**
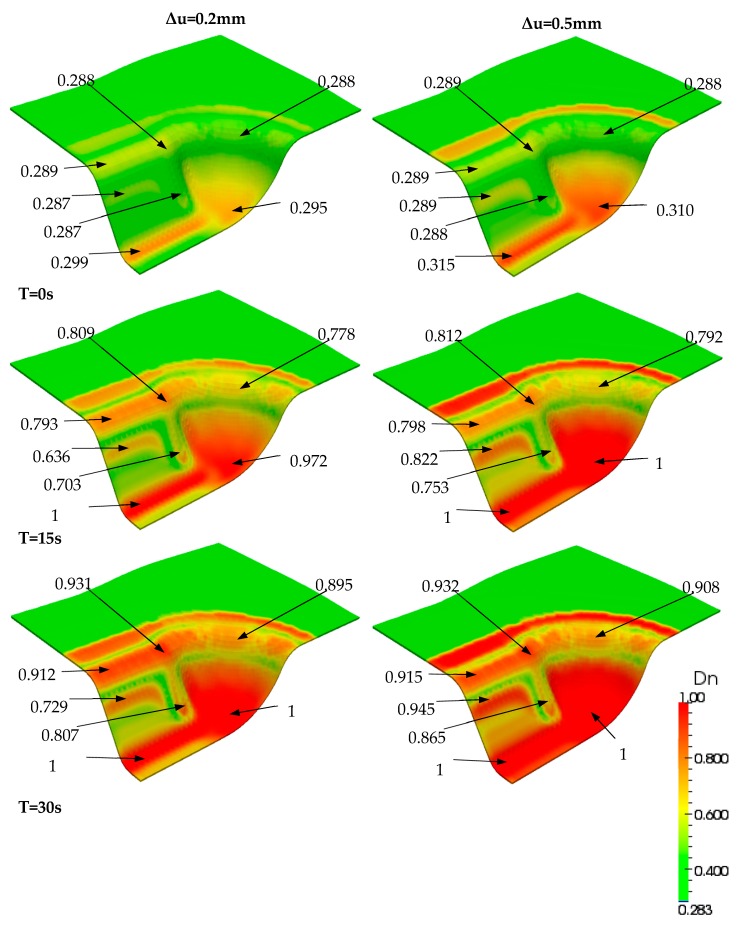
Comparison between two simulation results under a compaction displacement of Δu = 0.2 mm (left) and Δu = 0.5 mm (right) during the first 30 s.

**Figure 14:**
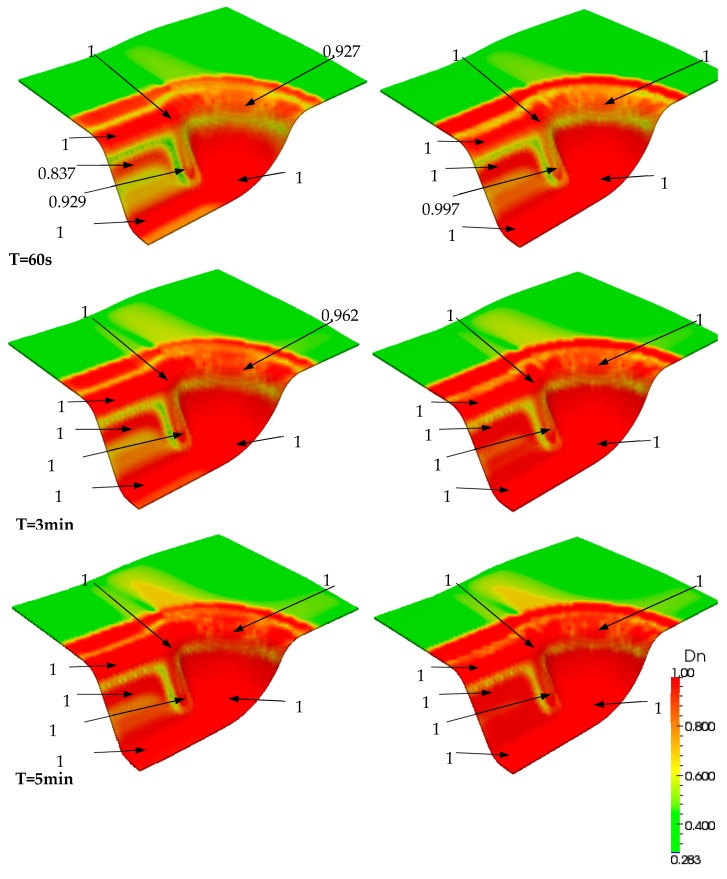
Comparison between two simulation results under a compaction displacement of Δu = 0.2 mm (left) and Δu = 0.5 mm (right) after 30 s up to 5 min.

Finally, the consolidation evolved within the final compaction load that was maintained. The developments over time in the middle face of the part are displayed in Figure 13 and Figure 14, according to the two compaction levels. The figures reveal that increased time leads to higher consolidation levels. The test with a compaction displacement of 0.5 mm required about 3 min of consolidation to obtain a relatively complete consolidation level through the active region of the part. However, more than 5 min were needed when the consolidated displacement was 0.2 mm. Furthermore, designers could obtain more guidance for industrial manufacturing by assessing the consolidation level through the part, which would make it possible to optimize the processing parameters and thereby the geometry design.

### 5.4. Remark Concerning the Type of Element and The Finite Element Software

Solid-shell elements show a good performance in the case of both thin parts (equivalent to that of standard shell finite elements) and deformation/stress in thickness calculations. The 7-node prismatic element briefly presented in Section 5.1 provides thickness stresses that verify the load boundary conditions on the upper and lower surfaces, and is essential for the analysis of the consolidation. The viscoelastic law and the calculation of the intimate contact are implemented in this prismatic element. This would not be possible in standard shell finite elements that are classically based on a plane stress assumption.

The viscoelastic constitutive law and the calculation of the intimate contact can be implemented in 3D elements of finite element codes using user subroutines such as UMAT/VUMAT for the ABAQUS software [99]. However, this approach will only be numerically effective in the case of composites that are thick enough, otherwise 3D finite elements are not efficient.

Another possibility for using the approach proposed for the consolidation calculation in a commercial finite element code is to implement a solid-shell element that performs an efficient calculation of the stress in the thickness (such as the prism that has been presented). This can be done in the ABAQUS software by creating a UELEM/VUELEM user subroutine that makes it possible to develop a specific element.

## 6. Conclusions

In summary, modeling of the consolidation behavior is crucial to assess void defects during thermoforming of thermoplastic composite prepregs. In this context, a viscoelastic model based on the generalized Maxwell approach was proposed by considering the elastic fiber bed and the viscosity of the thermoplastic matrix. The 3D constitutive behavior was accounted for in a hyperelastic framework. Five major deformation modes, i.e., two elongations in the warp and weft directions, in-plane shearing deformation, bending deformation, and transverse compression deformation, were highlighted separately. Among them, the viscous response was not only addressed for the in-plane shearing behavior but also for the compaction. As a result, the hyperelastic potential was decomposed by characterizing each physical invariant related to its deformation mode. Moreover, a time-discretized approach was realized for the intimate contact model, which makes the evolution of the consolidation level evident, and helps optimize the process conditions (pressure, temperature and time), which leads to better control of the process. In order to calibrate the parameters of the studied material associated with the considered models, a compaction test at a high temperature was performed. The proposed models were integrated into a finite element software, and the recently developed solid-shell finite element approach was used to simulate the compaction test and the thermoforming processing of a double dome within the consolidation part. The simulation results revealed that a good agreement was found with experimental data. Consequently, the evolution of the consolidation during simulation of the consolidation step made it feasible to achieve a complete consolidation level though the final product by controlling the processing parameters.

The cooling phase at the end of the process is of great importance. Crystallization was not directly addressed in this work. We proposed a method of simulation of the thermoforming process and the calculation of a consolidation indicator (degree of intimate contact). In this study, we focused on the simulation of the consolidation, it will be important to add additional software to analyze and simulate the crystallization during the cooling phase.

## Figures and Tables

**Table 1:** Main material properties.

Matrix	PA 6-6
**Fibers**	Carbon
**Weaving pattern**	2 × 2 twill
**Number of layers**	4
**Thickness (mm)**	2.05
**Surface density (g.mm-2)**	2600
**Fiber volume content**	0.6

**Table 2:** Optimized parameters related to consolidation deformation at different temperatures.

	260 °C	270 °C	280 °C	300 °C	Units
**D1**	2.274 × 10^−2^	6.814 × 10^−2^	2.177× 10^−2^	8.793 × 10^−2^	[J mm^−2^]
**D2**	4.113 × 10^−1^	5.870× 10^−1^	3.607× 10^−1^	6.889× 10^−1^	[J mm^−2^]
**D3**	2.478	1.080	1.279	1.466	[J mm^−2^]
**D4**	5.386	3.434 × 10^-1^	1.974	-3.411	[J mm^−2^]
**D5**	4.170	1.383	−9.312 × 10^−1^	6.299 × 10^−1^	[J mm^−2^]
**γ1**	7.293	1.043	5.236 × 10^−1^	8.024 × 10^−1^	-
**γ2**	4.499 × 10^−1^	5.754 × 10^−1^	9.420 × 10^−2^	4.443 × 10^−1^	-
**τ1**	35.87	8.473 × 10^−1^	5.787 × 10^−2^	3.638 × 10^−1^	[s]
**τ2**	20.37	6.724 × 10^−1^	3.262 × 10^−1^	3.881 × 10^−1^	[s]

**Table 3:** Optimized parameters related to the intimate contact model for a 2 × 2 twill in carbon/PA 6-6 prepregs at different temperatures.

	260 °C	270 °C	280 °C	300 °C	Units
**k1**	1.85				-
**k2**	0.82				-
***η***	$\eta =6.15\times 10^{-16}\times \exp\left(\frac{21901}{T\left(K\right)}\right)$				[MPa·s]

## Appendix A. Strain Energy Functions Associated with Each Deformation Mode

### A.1. Elongation Strain Energy

The studied reinforcement of a prepreg material is balanced: the mechanical behavior during tension is identical in both the warp and weft directions. For the sake of simplicity, a second-order polynomial function is defined for the strain potential energy:

(A1) $$w_{elongi}=T_{i}{\left(I_{elongi}\right)}^{2}\;\;i=1,2$$

where $T_{i}$ is the tensile stiffness in the direction of the yarn i. Identification of the parameters can be obtained both by an approximate analytical homogenization approach and a tensile test at a high temperature.

### A.2. Bending Strain Energy

For thermoplastic prepregs, a bending behavior with a strong temperature dependence is used, while the viscosity of the bending behavior is ignored. The strain energy linked to the bending invariant is written as follows:

(A2) $$w_{ben}=B_{1}(T){\left(I_{\chi 1}\right)}^{2}+B_{2}(T){\left(I_{\chi 2}\right)}^{2}$$

where the two thermo-dependent coefficients B1 and B2 are identical on account of the balance of the material. They can be obtained from experimental results by cantilever tests at a high temperature.

### A.3. In-plane Shear Strain Energy

The strain energy density expression is usually described by a polynomial, piecewise of its physical invariant related to the shear angle. By considering the presence of the matrix for thermoplastic prepreg composites, the viscoelastic model with thermo-dependence is introduced here. This model is based on the generalized Maxwell model, a free energy function is completed by the effect of viscoelastic internal variables $\underline{\underline{Q_{i}}}$:

(A3) $$\psi (\underline{\underline{C}},\underline{\underline{Q_{i}}})=w_{shear}(I_{shear})-\frac{1}{2}\sum_{i=1}^{N}(\underline{\underline{C}}:\underline{\underline{Q_{i}}})$$

where the first part $w_{shear}$ is the in-plane shear energy, also called the free energy at equilibrium, i.e., the energy stored after all viscous effects have ended. The second term characterizes the non-equilibrium state.

For the equilibrium part, a polynomial function with thermo-dependence is introduced as:

(A4) $$w_{shear}\left(I_{shear},T\right)=\left(\alpha \;T+\beta \right)\cdot \sum_{i=2}^{8}C_{i}I_{shear}^{i}\;{250\;}^{o}C<T\leq {300\;}^{o}C$$

All of the coefficients for an elastic equilibrium part and the internal variables for the viscous relaxation part can be identified by a bias-extension test at a high temperature [100].

## Appendix B. List of Symbols

**Nomenclature**			
*List of definition*			
$\underset{=}{S}$/**S**	Second Piola-Kirchoff stress tensor	P_app_	Applied pressure
$\underset{=}{C}$/**C**	Right Cauchy-Green strain tensor	a, b, w	Dimension parameters
w	Internal potential energy	D	Degree of intimate contact
$\underset{=}{F}$/**F**	Deformation gradient tensor	η	Matrix viscosity
I_1_, I_2_, I_3_… I_n_	Invariants	k_1_, k_2_	Geometric ratios
A_elong1_, A_elong2_	Parameter A related to elongation mode in warp, weft direction	A_0_, A_n_, A_n+1_	Parameter A at initial state, before, after n^th^ time increment
A_χ1_, A_χ2_	Parameter A related to bending mode in warp, weft direction	A_shear_, A_comp_	Parameter A related to in-plane shearing mode, and compression mode
T	Temperature	$W_{\mathrm{ext}},{\;W}_{\mathrm{int}},{\;W}_{\mathrm{acc}}$	External, internal and inertial work
E_i_	Young’s modulus of spring element i	δu	Virtual displacement vector
$\eta_{i}$	Viscosity of dashpot i	**u**, $\ddot{u}$	Displacement and acceleration vectors
τ_i_	Relaxation time	$F_{\mathrm{ext}},\;F_{\mathrm{int}}$	Exterior and interior load vectors
γ_i_	Relative weight	Superscript α	Index related to bending, membrane, or pinching deformation
$\underset{=}{Q}$	Stress-like internal variable	**σ**	Cauchy stress tensor
$\underset{=}{H}$	Algorithmic solution of Q	**B**	Strain interpolation matrix
D_k_, C_i_	Polynomial parameters	Ω_e_	Volume of element
ψ	Free energy function	T_1_, T_2_	Tensile stiffness in two directions of yarn
t_n_, t_n+1_	Times at the beginning and end of the n^th^ time increment	M_χ1_, M_χ2_	Bending moments in two directions of yarn
Δt_n_	n^th^ time increment	B_1_, B_2_	Material parameters related to bending
		α, β	Thermo-dependent parameters
